# P03-002 - Different phenotypes associated with Q703K variant

**DOI:** 10.1186/1546-0096-11-S1-A197

**Published:** 2013-11-08

**Authors:** A von Scheven-Gête, I Moix, G Simon, N Busso, M Morris, M Hofer

**Affiliations:** 1 Centre Hospitalier Universitaire Vaudois, Lausanne, Switzerland; 2 Hopitaux Universitaires Genevois, Geneve, Switzerland

## Introduction

PFAPA syndrome is characterized by recurrent fever with aphtosis, pharyngitis and cervical adenitis. It is suspected to be an auto-inflammatory disease (AID). For some AID, a monogenic origin has been established in the past years. In PFAPA no clear aetiology has been found yet. However in some patients variants in the NLRP3 gene can be found. The variant Q703K was described so far in patients with CAPS as well as healthy carriers.

## Objectives

To describe the phenotype of our patients with recurrent fever presenting the NLRP3 variant Q703K.

## Methods

In patients presenting to our consultation with periodic fever suspected to be auto-inflammatory, we screened genomic DNA by PCR and sequenced for genetic variants of NLRP3 genes. The symptoms, treatment, response to treatment and family history of the patients with the variant Q703K have been retrospectively extracted and described.

## Results

We found the NLRP3 variant Q703K in 11 patients. Ten were PFAPA patients among 97 patients from our cohort and one had a CAPS phenotype: 8 boys and 3 girls with a median age of 12 months at disease onset and a median age of 52 months at diagnosis. In the PFAPA patients, family history was positive for febrile episodes or for tonsillectomy in 6 patients. The median duration of fever was 4 days and the median interval was 4 weeks. Pharyngitis was always present in 6 patients and in most episodes in 3 patients. Cervical adenitis presented in every episode in 6 patients, in most episodes in 2 patients and rarely in 1 patient. Aphtosis was found only in 1 patient in every episode, in 6 patients sometimes and in one patient in most episodes. 5 patients expressed abdominal pain that accompanied most fever episodes. 1 patient showed sometimes arthralgia, 2 patients had headaches in most episodes and one patient had once a cutaneous rash. All patients were well and without symptoms in between febrile episodes. 5 out of 7 patients treated by corticosteroids responded promptly. In the other two patients two doses were often necessary. 3 patients underwent tonsillectomy: one with no effect, in 2 the fever episodes resolved but one patient had persistent episodes of aphtosis. In 4 patients genomic sequencing of the parents was done; one parent positive for Q703K had a history of recurrent febrile episodes, but the 3 other parents did not present a history of recurrent fever episodes nor recurrent pharyngitis nor tonsillectomy. The patient with CAPS phenotype presented with urticarial rash, partial deafness, arthralgias and elevated inflammatory parameters.

## Conclusion

11 of our patients presented with the variant Q703K but 10 had clearly a phenotype of PFAPA and only one the phenotype of CAPS. This could suggest that variants in fever genes can be associated with different phenotypes and that probably more than one gene could be implicated in the pathogenesis.

## Competing interests

None Declared.

